# A new trapdoor spider of *Cyclocosmia* Ausserer, 1871 from southern China (Araneae, Halonoproctidae)

**DOI:** 10.3897/BDJ.11.e98311

**Published:** 2023-04-07

**Authors:** Kun Yu, Changze Li, Shuyuan Zhang, Feng Zhang

**Affiliations:** 1 Key Laboratory of Zoological Systematics and Application of Hebei Province, Institute of Life Science and Green Development, College of Life Sciences, Hebei University, Baoding, China Key Laboratory of Zoological Systematics and Application of Hebei Province, Institute of Life Science and Green Development, College of Life Sciences, Hebei University Baoding China; 2 College of Marine Sciences, South China Agricultural University, Guangzhou, China College of Marine Sciences, South China Agricultural University Guangzhou China; 3 Shanghai FlyDreamer Cultural Diffusion Co.,Ltd, Shanghai, China Shanghai FlyDreamer Cultural Diffusion Co.,Ltd Shanghai China

**Keywords:** Opisthosomal disc, early stages of spiderling, morphology, taxonomy, variation

## Abstract

**Background:**

The genus *Cyclocosmia* Ausserer, 1871 previously included ten species from North America and Asia, six of which have been recorded from China.

**New information:**

A new species, *Cyclocosmiaruyi* Yu & Zhang **sp. n.**, is described and diagnosed, based on both sexes from Guangxi Province, China. Morphological characters for the early stages of juveniles of the new species are also provided.

## Introduction

Trapdoor spiders of the genus *Cyclocosmia* Ausserer, 1871, are currently known from Southeast Asia (south China, Thailand, Vietnam and Laos) and the coast of the Gulf of Mexico (southern United States and Mexico) (World Spider Catalog 2023). These spiders are famous in their unique opisthosomal discs bearing orderly ribs and grooves, as well as exquisite muscle impressions. They show interesting defence behaviour of their unusual burrow, which is composed of a wide vestibule and a narrow basal tube (Schwendinger 2005: 225). When disturbed, spiders will commonly retreat to the lower portion of their burrow and use the opisthosomal disc as a false bottom. They are relatively rare in collections and previous studies have shown that this rarity is due to both their demanding requirements of habitat and the well-camouflaged trapdoor that is difficult to find (Gertsch and Platnick 1975). However, in some areas, they are locally abundant with relatively high density (Schwendinger 2005: 240).

Previous studies indicated *Cyclocosmia* are very long-lived spiders like some other mygalomorphs, with more than 12 years of life span and taking at least five years to become mature (Gertsch and Platnick 1975: 2, Schwendinger 2005: 250). Some species do not reproduce annually; they potentially take a full year to brood eggs and can reproduce more than 300 spiderlings one time (Schwendinger 2005: 240, 250). Wild-caught spiderlings with fully developed opisthosomal discs were considered at least in the third instar (Schwendinger 2005: 237). However, no complete record for the early stages of juvenile *Cyclocosmia* has been documented and the life history of *Cyclocosmia* is still poorly known in some respects.

**Figure 1.**
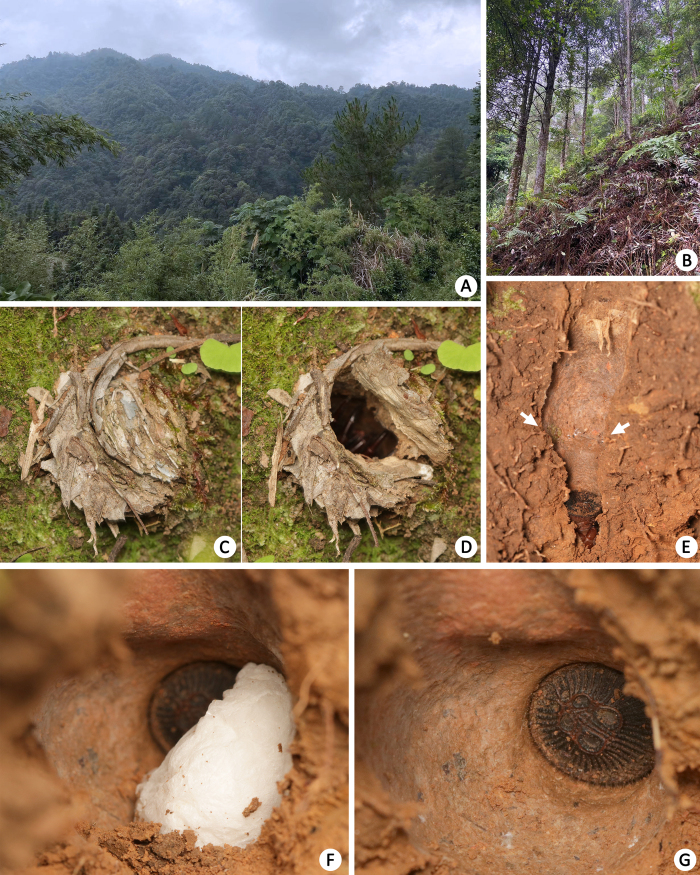
Habitats (**A–B**) and burrows (**C–E**) of *Cyclocosmiaruyi* Yu & Zhang **sp. n**. **A** Landscape of type locality; **B** Microhabitat; **C–D** Burrow of holotype female, trapdoor closed (**C**) and opened (**D**); **E** Profile of burrow of paratype female (MHBU-ARA-00023657), with arrows indicating constricted basal tube; **F–G** Holotype female using opisthosomal disc as the false bottom of burrow, with egg sac (**F**) and egg sac removed (**G**). Figures are copyright 2023 Kun Yu.

**Figure 2.**
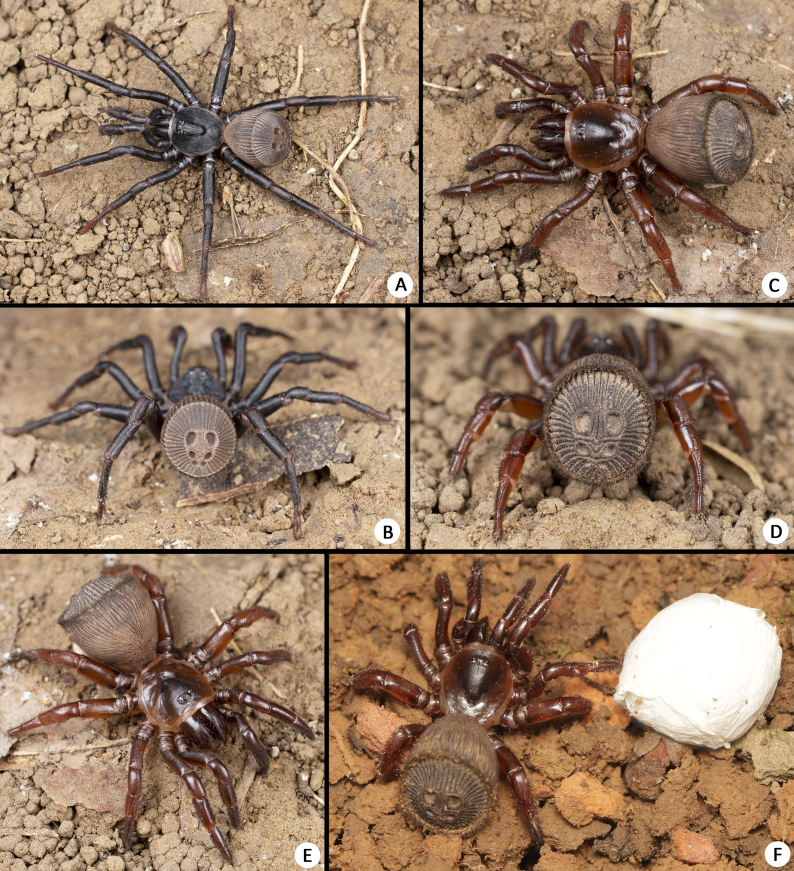
Living specimens of *Cyclocosmiaruyi* Yu & Zhang **sp. n.**. **A–B** Paratype male (MHBU-ARA-00023660); **C–F** Holotype female. **A–E** Copyright 2023 Weihang Wang; **F** Comparison of female and egg sac, copyright 2023 Kun Yu.

**Figure 3.**
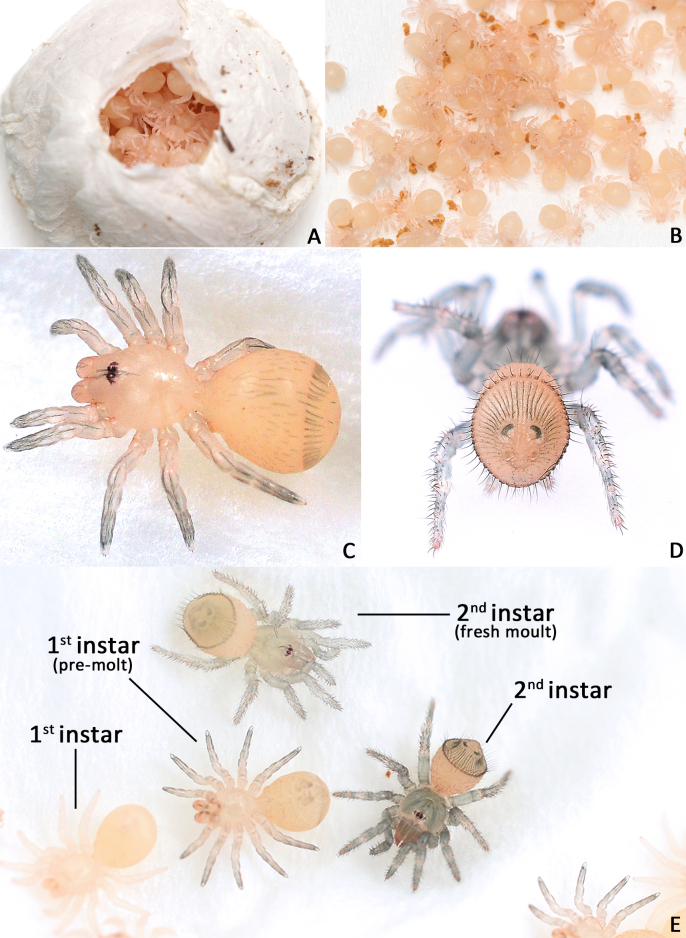
Living spiderlings of *Cyclocosmiaruyi* Yu & Zhang **sp. n**. **A** First instar spiderlings in egg sac; **B** first instar spiderlings, with eggshells; **C** Late stage of a first instar spiderling, pre-moult; **D** Second instar spiderlings, back view; **E** Comparison.

In this study, we describe a new species of *Cyclocosmia*, based on both sexes from Guangxi Province, China, herein named *Cyclocosmiaruyi* Yu & Zhang, sp. n. During the survey of this new species in 2022, we obtained its egg sacs and brought them into the laboratory for observations and rearing (Figs. 1–3; see under Biology). The early stages of juveniles of the new species are preliminarily reported and the opisthosomal disc of the new species is confirmed to develop in the second instar.

## Materials and methods

All specimens, preserved in 75–95% ethanol, were examined under a ZEISS Stemi 305 stereomicroscope. The photographs of genitalia were taken by an Olympus BX53 microscope equipped with a Kuy Nice CCD Camera. Photographs of specimens were stacked by the Helicon Focus 7 software and retouched in the Adobe Photoshop CC 2019 software. Specimens were measured in millimetres by the dedicated tool of Leica LAS V. 4.3 software. The measurements of palps are shown as: total length (femur, patella, tibia, tarsus) (male palpal tarsus measured cymbium only); measurements of legs are shown as: total length (femur, patella, tibia, metatarsus, tarsus). Leg formulae are arranged from longest to shortest. Spinations are given as left/right or a single number, if no variation was observed between both sides. Female vulvae were cleared with Pancreatin (BBI Life Sciences). Distributions of Asian *Cyclocosmia* spp. were mapped using ArcMap 10.5 (Esri Inc.). All specimens studied are deposited in the Museum of Hebei University, Baoding, China (MHBU) or Collection of Kun Yu, Hanzhong, China (CKYH).

Cytochrome c oxidase subunit 1 (COI) sequences of new species and five other *Cyclocosmia* species (*C.lannaensis*, *C.latusicosta*, *C.ricketti*, *C.sublatusicosta* and *C.subricketti*) were amplified using universal primers LCO1490 (forward) and HCO2198 (reverse) (Folmer et al. 1994). PCR products were sent to Azenta Life Sciences, Inc. (Tianjin, China) for sequencing. Sequences were uploaded to the National Center for Biotechnology Information (NCBI) and GenBank accession numbers and voucher information are shown in Table 1 (data released from 5 April 2023). COI sequences of *C.truncata* and *C.loricata* were downloaded from NCBI (GenBank accession numbers as shown in Table 1). Sequences were imported into MEGA-X (Kumar et al. 2018) for multiple alignment and calculation of genetic distances. Three species of *Cyclocosmia* (*C.siamensis* Schwendinger, 2005, *C.liui* Xu, Xu & Li, 2017 and *C.torreya* Gertsch & Platnick, 1975) were not included in our molecular analysis due to lacking specimens or accessible data in the public database.

**Abbreviations**: ALE: anterior lateral eyes, AME: anterior median eyes, PLE: posterior lateral eyes, PME: posterior median eyes, MOA: median ocular area, d: dorsal, pd: prodorsal, pl: prolateral, pv: proventral, rd: retrodorsal, rl: retrolateral, rv: retroventral, v: ventral.

**Comparative material examined**

*Cyclocosmialannaensis* Schwendinger, 2005: 1 female and 1 juvenile (CKYH), CHINA: Yunnan Province, Xishuangbanna, Menglun Town, Xishuangbanna Tropical Botanical Garden, 21.8794°N, 101.3293°E, 10 Apr 2019, leg. C. Wei; 2 males and 2 juveniles (CKYH), CHINA: Yunnan Province, Xishuangbanna, Jinghong, Mt. Jinuo, 22.0636°N, 101.0054°E, 5 July 2022, leg. X. Yang (males were raised and matured in Dec 2022); 2 females (CKYH), CHINA: Yunnan Province, Xishuangbanna, Puwen Town, near Mengwang Village, 22.4830°N, 101.2549°E, 6 Oct 2022, leg. G. Qi.

*Cyclocosmialatusicosta* Zhu, Zhang & Zhang, 2006: 1 female (Holotype; MHBU), CHINA: Guangxi Province, Ningming County, Aidian Town, 25 Aug 2004, leg. M. Zhu, J. Zhang & F. Zhang; 2 females (Paratypes; MHBU), CHINA: Guangxi Province, Ningming County, Aidian Town, 25 Aug 2004, leg. M. Zhu, J. Zhang & F. Zhang; 1 male and 3 females (CKYH), VIETNAM: near Lào Cai, no further data.

*Cyclocosmiaricketti* (Pocock, 1901): 1 female (MHBU), CHINA: Zhejiang Province, Wenzhou, Taishun County, 27.56°N, 119.71°E, leg. Z. Chen, Dec 1989; 1 male (CKYH), CHINA: Zhejiang Province, Wenzhou, Mt. Yandang, near Nengren Temple, 28.3469°N, 121.0680°E, 15 Oct 2017, leg. S. Zheng; 1 male (CKYH), CHINA: Zhejiang Province, Wuchaoshan National Forest Park, Hangzhou, Mt. Xiaohe, 30.2076°N, 120.0389°E, 25 Oct 2017, leg. S. Zhang; 1 male (CKYH), CHINA: Zhejiang Province, Hangzhou, Wuchaoshan National Forest Park, near Lishanqiao Village, 30.2014°N, 119.9994°E, Sep 2016, leg. Y. Zhang; 1 female (CKYH), CHINA: Jiangxi Province, Ganzhou, Dayu County, near Wudong Village, 25.3951°N, 114.0271°E, 745 m elev., Oct 2021, leg. R. Kong.

*Cyclocosmiasublatusicosta* Yu & Zhang, 2018: 1 female (CKYH), CHINA: Guangdong Province, Foshan, Lubao County, Changqi Village, 23.3719°N, 112.9849°E, 3 Mar 2021, leg. X. Zhang; 1 male and 5 females (CKYH), CHINA: Guangxi Province, Qinzhou, Pubei County, 15 Apr 2020, leg. Q. Chen.

*Cyclocosmiasubricketti* Yu & Zhang, 2018: 1 female (CKYH), CHINA: Chongqing, Beibei district, Jindao Gorge, 30.0248°N, 106.6236°E, 12 Apr 2021, leg. H. Chen; 1 male, 3 females and 1 juvenile (CKYH), CHINA: Sichuan Province, Chengdu, citrus plantations in Pujiang County, 30.28°N, 103.56°E, 10 June 2017, leg. local collecter (male was raised and matured in July 2017); 1 female (CKYH), CHINA: Sichuan Province, Qionglai, near Shiqima Village, 30.3258°N, 103.7382°E, 20 Nov 2017, leg. B. Xu; 2 females (CKYH), CHINA: Sichuan Province, Qionglai, Apr 2018, no further data; 1 male (MHBU), CHINA: Sichuan Province, Leshan, Mt. Emei, near Shenshuige, 29.5709°N, 103.4182°E, 24 Sep 2010, leg. Y. Zhao & Z. Gao; 1 male (CKYH), CHINA: Hubei Province, Yichang, Changyang County, Duzhenwan Town, near Yangzheping Village, 30.3075°N, 110.9952°E, 22 Aug 2018, leg. H. Deng.

## Taxon treatments

### Cyclocosmia ruyi

Yu & F. Zhang sp. n.

E2550426-9D37-5B02-97CE-EAE86EFDF496

663698A3-0485-4445-846A-A5718D5BFA0C

#### Materials

- **Type status:** Holotype. **Occurrence:** recordedBy: K. Yu & Y. Ding; sex: female; occurrenceID: B26D100E-7810-5506-81EC-3FF0AD9AD810; **Taxon:** scientificName: *Cyclocosmiaruyi* Yu & Zhang, sp. n.; **Location:** country: China; stateProvince: Guangxi; county: Jinxiu; locality: Changdong Village, near Gubaotun; verbatimElevation: 980 m; verbatimCoordinates: 110.1668°E, 24.0970°N; **Identification:** identifiedBy: K. Yu; **Event:** eventID: HBUARA#2022-138; eventDate: 3 Aug 2022; **Record Level:** institutionCode: MHBU-ARA-00023656
- **Type status:** Paratype. **Occurrence:** recordedBy: K. Yu & Y. Ding; sex: 1 female; occurrenceID: 39128A4E-FE8F-592C-B347-587E66971A64; **Taxon:** scientificName: *Cyclocosmiaruyi* Yu & Zhang, sp. n.; **Location:** country: China; stateProvince: Guangxi; county: Jinxiu; locality: Changdong Village, near Gubaotun; verbatimElevation: 980 m; verbatimCoordinates: 110.1668°E, 24.0970°N; **Identification:** identifiedBy: K. Yu; **Event:** eventID: HBUARA#2022-138; eventDate: 3 Aug 2022; **Record Level:** institutionCode: MHBU-ARA-00023657
- **Type status:** Paratype. **Occurrence:** recordedBy: W. Feng; sex: 1 male, 1 female; occurrenceID: 610035A4-763C-5D07-96BD-476A82FC3241; **Taxon:** scientificName: *Cyclocosmiaruyi* Yu & Zhang, sp. n.; **Location:** country: China; stateProvince: Guangxi; county: Jinxiu; locality: Changdong Village, near Gubaotun; verbatimElevation: 980 m; verbatimCoordinates: 110.1668°E, 24.0970°N; **Identification:** identifiedBy: K. Yu; **Event:** eventDate: 7 Oct 2022; **Record Level:** institutionCode: MHBU-ARA-00023658~00023659
- **Type status:** Paratype. **Occurrence:** recordedBy: W. Feng; sex: 1 male (raised by C. Li and matured on 1 Oct 2022); occurrenceID: 06EAD122-F12C-5D31-91A6-9B73D04E142A; **Taxon:** scientificName: *Cyclocosmiaruyi* Yu & Zhang, sp. n.; **Location:** country: China; stateProvince: Guangxi; county: Jinxiu; locality: Changdong Village, near Gubaotun; verbatimElevation: 980 m; verbatimCoordinates: 110.1668°E, 24.0970°N; **Identification:** identifiedBy: K. Yu; **Event:** eventDate: 29 June 2022; **Record Level:** institutionCode: MHBU-ARA-00023660
- **Type status:** Other material. **Occurrence:** recordedBy: W. Feng; sex: 1 male (penultimate); occurrenceID: D52EA05E-D9A1-5B54-A681-5608F39BA65E; **Taxon:** scientificName: *Cyclocosmiaruyi* Yu & Zhang, sp. n.; **Location:** country: China; stateProvince: Guangxi; county: Jinxiu; locality: Changdong Village, near Gubaotun; verbatimElevation: 980 m; verbatimCoordinates: 110.1668°E, 24.0970°N; **Identification:** identifiedBy: Kun Yu; **Event:** eventDate: July 2017; **Record Level:** institutionCode: CKYH (DNA voucher number: KYU081)
- **Type status:** Other material. **Occurrence:** recordedBy: W. Feng; sex: 2 females (1 adult & 1 subadult); occurrenceID: ED50D521-B57C-5E45-9B27-22057CFBEE25; **Taxon:** scientificName: *Cyclocosmiaruyi* Yu & Zhang, sp. n.; **Location:** country: China; stateProvince: Guangxi; county: Jinxiu; locality: Changdong Village, near Gubaotun; verbatimElevation: 980 m; verbatimCoordinates: 110.1668°E, 24.0970°N; **Identification:** identifiedBy: Kun Yu; **Event:** eventDate: July 2017; **Record Level:** institutionCode: CKYH (the subadult female were used for DNA extraction, DNA voucher number: KYU082)

#### Description

**Female (Holotype, MHBU-ARA-00023656).** Sclerotised parts of body mostly reddish-brown, membranes cream, cephalon and fovea slightly darker, opisthosoma yellowish-brown, but gradually darkens posteriorly, opisthosomal disc dark brown (Fig. 5C). Colour slightly darker in life, especially cephalon and chelicerae (Fig. 2C–F). Total length (not including chelicerae) 18.48. Carapace smooth, 7.93 long, 7.37 wide. Eyes on low mound, eye group 0.79 long, 2.10 wide anteriorly, 2.09 wide posteriorly. Eye diameters and interdistance: AME 0.24, ALE 0.43, PME 0.20, PLE 0.30, ALE–AME 0.36, AME–ALE 0.41, PME–PME 0.18, PME–PLE 0.21, ALE–PLE 0.15, MOA 0.84 long, front width 0.85, back width 1.47. Chelicerae robust, promargin of cheliceral groove with 12 and retromargin with 10 denticles of different sizes, arranged in irregular rows. Rastellum carrying one retrolateral-proximal spine and ca. 10 distal spines. Maxillae 3.28 long, 2.06 wide, carrying 12/9 cuspules in prolateral-proximal corner and many weaker spicules all over ventral surface. Labium 1.43 long, 2.00 wide, carrying two cuspules. Sternum 5.47 long. 4.83 wide, three pairs of sigilla present, two anterior pairs small, anterior one closer to margin than median one, posterior pair of sigilla large, medially fused.

Measurements of palp: 13.95 (5.28, 2.41, 3.05, 3.21). Legs relatively short and robust, without scopula. Measurements of legs: I 15.98 (5.68, 2.78, 3.59, 2.34, 1.59), II 14.02 (4.92, 2.27, 2.94, 2.18, 1.71), III 13.65 (4.79, 2.59, 2.04, 2.35, 1.88), IV 16.59 (5.39, 2.73, 2.92, 3.24, 2.31). Leg formula 4213. Palpal claw with one proximal tooth on common base and one denticle slightly closer to front base of that tooth, the tooth with one small denticle each front and back surface. Each claw of leg tarsus with one small denticle and one larger tooth near base. Spination: Palp, patella 1 pv distal spine, tibia pv 36/33, rv 35/37, tarsus pv 41/42, rv 57/50; legs I, tibia pv 29/22, rv 39/31, metatarsus pv 40/38, rv 44/35, tarsus pv 27/26, rv 19/21; II, tibia pv 14/11, rv 11/15, metatarsus pv 34/30, rv 19/16, tarsus pv 25/23, rv 10/11; III–IV with many pd and distodorsal spines on patellae and tibiae, d and pd spines on metatarsus III and pd spines on metatarsus IV, 5/4 pd distal spines on metatarsus III, 12/14 pd to ventral distal spines on tarsus III, some of spines on Legs III–IV difficult to distinguish from stiff bristles.

Trichobothria: Palp, tibia with 3 pd, 2 rd in proximal half, tarsus with 8/9 pd (one of trichobothrial sockets carrying two trichobothria on right metatarsus), arranged on irregular oblique row; Legs I, tibia with 4 pd, 4 rd in proximal half, metatarsus with 6 d in distal half, tarsus with 13/14 d, irregularly arranged; Legs II–IV share similar trichobothrial position and arrangement to I; II, tibia 4 pd, 4 rd, metatarsus 6/5 d, tarsus 14/15 d; III, tibia 3/4 pd, 3 rd, metatarsus 6/5 d, tarsus 14/18 d; IV, tibia 5 pd, 5 rd, metatarsus 5 d, tarsus 9 d.

**Figure 4.**
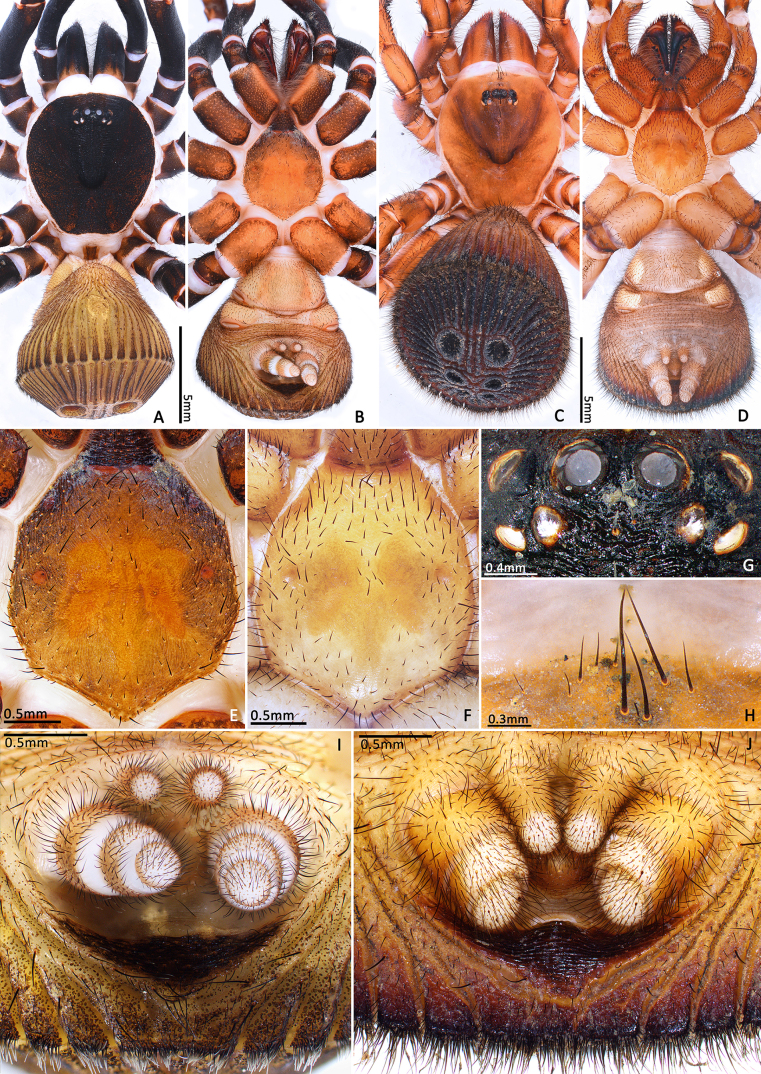
*Cyclocosmiaruyi* Yu & Zhang **sp. n.**. **A–B, E, G, I** Paratype male (MHBU-ARA-00023660); **C–D, H** Paratype female (MHBU-ARA-00023657); **F** Paratype female (MHBU-ARA-00023659); **J** Holotype female; **A–D** Habitus; **E–F** Sternum; **G** Ocular area; **H** Bristles on clypeus; **I–J** Spinnerets. **A, C, G–H** Dorsal view; **B, D, E–F, I–J** Ventral view.

**Figure 5.**
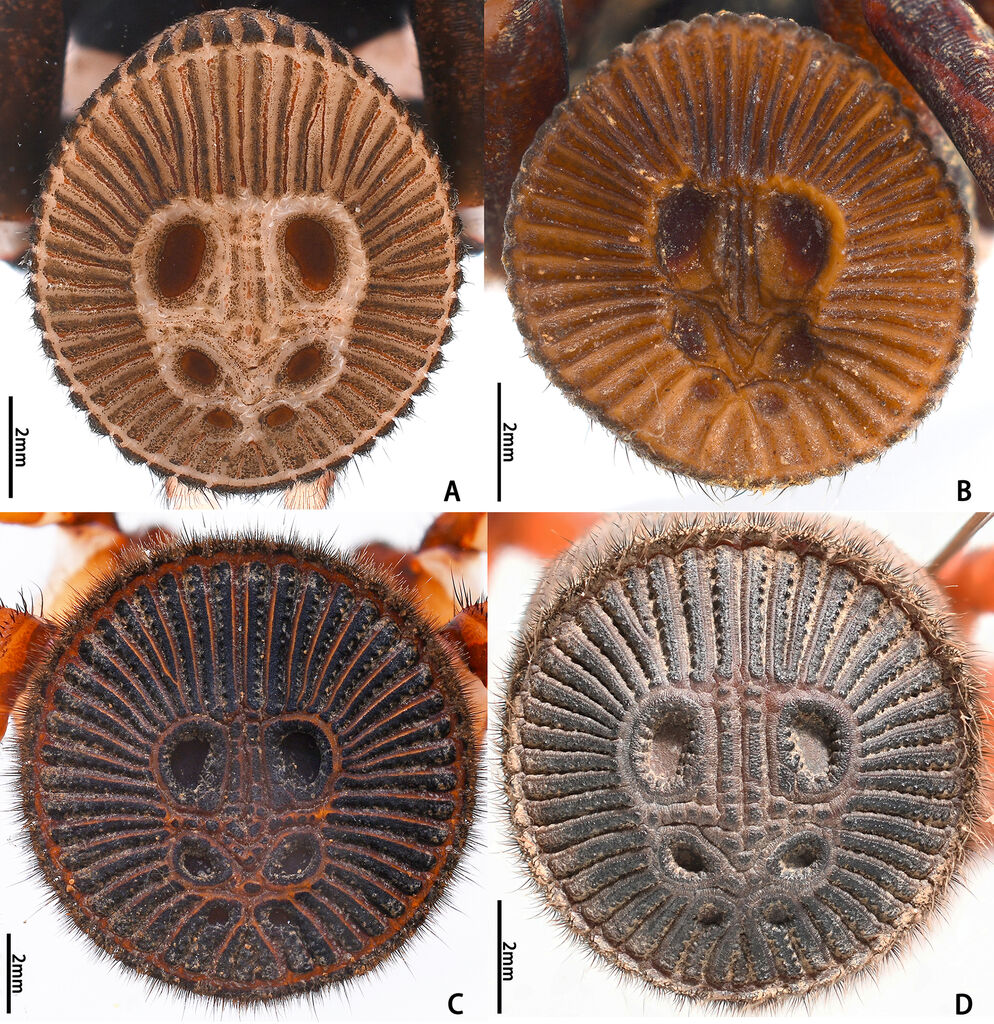
Opisthosomal discs of *Cyclocosmiaruyi* Yu & Zhang **sp. n.**. **A** Paratype male (MHBU-ARA-00023660); **B** Paratype male (MHBU-ARA-00023658); **C** Holotype female; **D** Paratype female (MHBU-ARA-00023659).

Opisthosoma 9.15 long. Opisthosomal disc 11.33 in diameter, carrying ca. 35 bristles on each rib angle, with three pairs of muscle impressions centrally, two longitudinal ribs separating upper muscle impressions from each other, two transversal ribs separating upper and median pair of muscle impressions, ends of lower transversal rib not connected to radial ribs, ribs between muscle impressions slightly fragmentised; with 45 radial ribs around muscle impressions, each rib carrying tubercles in one row and many adjacent smaller rough granulations irregularly arranged besides the tubercles; no setae present on disc surface, except for three pairs of bristles on rims of muscle impressions (upper pair on inner lower corner of rim of upper muscle impressions, median pair on inner median part of rim of median muscle impressions and lower pair on inner upper part of rim of lower muscle impressions) (Fig. 5C). Posterior median spinnerets 1.25 long, posterior lateral spinnerets 3-segmented (Fig. 4J), 2.15 long.

Spermathecae long, distally curved inwards, ental margin concave near mid-point; with dense pores (Fig. 7A, 9C).

**Male (Paratype, MHBU-ARA-00023660).** Sclerotised parts of body mostly reddish-black, membranes cream, leg tarsi slightly lighter than other segments (Fig. 2A–B). Opisthosoma yellowish-brown, ribs slightly darker (Fig. 4A–B). Total length (not including chelicerae) 16.50. Carapace 7.03 long, 6.29 wide, rather rough, with some transverse ridges on cephalon and dense pits. Eyes on low mound (Fig. 4G), eye group 0.78 long, 1.98 wide anteriorly, 1.90 wide posteriorly. Eye diameters and interdistance: AME 0.44, ALE 0.42, PME 0.32, PLE 0.28, AME–AME 0.18, AME–ALE 0.26, ALE–PLE 0.24, PME–PME 0.82, PME–PLE 0.06, MOA 0.81 long, front width 1.03, back width 1.29. Chelicerae relatively weaker than female, promargin of cheliceral groove with 13/15 and retromargin with 11/10 denticles of different sizes. Rastellum carrying one retrolateral-proximal spine and 7/6 distal spines. Maxillae 3.16 long, 1.65 wide, carrying 10 cuspules in prolateral-proximal corner. Labium 1.07 long, 1.54 wide, carrying two cuspules. Sternum 5.47 long. 4.83 wide, with three pairs of sigilla (Fig. 4E) like female in position and shape.

Measurements of palp: 14.33 (5.58, 2.52, 4.51, 1.72). Legs relatively short and robust, all tarsi present scopula. Measurements of legs: I 22.14 (7.33, 2.60, 5.27, 4.66, 2.28), II 19.43 (6.12, 2.57, 4.27, 4.26, 2.21), III 18.18 (5.47, 2.69, 3.16, 4.20, 2.66), IV 23.30 (6.68, 3.31, 4.46, 5.84, 3.01). Leg formula 4123. Claws of leg tarsus like female. Leg spination: I, tibia pv to rv 19 (pv 3, distally), metatarsus pv to rv 20, tarsus pl to pv 14/13, v 1/2, rv 11/8; II, tibia pv to rv 19, metatarsus pv to rv 25, tarsus pl to pv 11/13, v 2/3, rv 20/21; III–IV with many spines (Fig. 10A), but none on dorsal and retrolateral sides of tibiae III–IV and retrolateral side of metatarsus IV, most spines on legs III–IV difficult to distinguish from stiff bristles.

Trichobothria: Palp, tibia with 5 d in proximal half, cymbium with 7/6 d in distal half, arranged in an irregularly oblique row; Legs I, tibia with 4 pd, 4 rd in proximal half, metatarsus with 5 d in distal half, tarsus with 19/14 d, irregularly arranged; Legs II–IV share similar trichobothrial position and arrangement to I; II, tibia 4 pd, 4 rd, metatarsus 5/6 d, tarsus 16/14 d; III, tibia 4/3 pd, 4 rd, metatarsus 3 d, tarsus 17/15 d; IV, tibia 4 pd, 4 rd, metatarsus 4/5 d, tarsus 8/7 d.

Opisthosoma 8.05 long, disc 7.94 in diameter, with ca. 30 bristles on each rib angle (Fig. 11A). Muscle impressions and ribs between muscle impressions as in female, but not strongly sclerotised; 45 radial ribs present (Fig. 5A), without tubercles. Bristles of disc like female in number and position. Posterior median spinnerets 1.00 long, posterior lateral spinnerets 3-segmented (Fig. 4I), 2.77 long.

**Figure 6.**
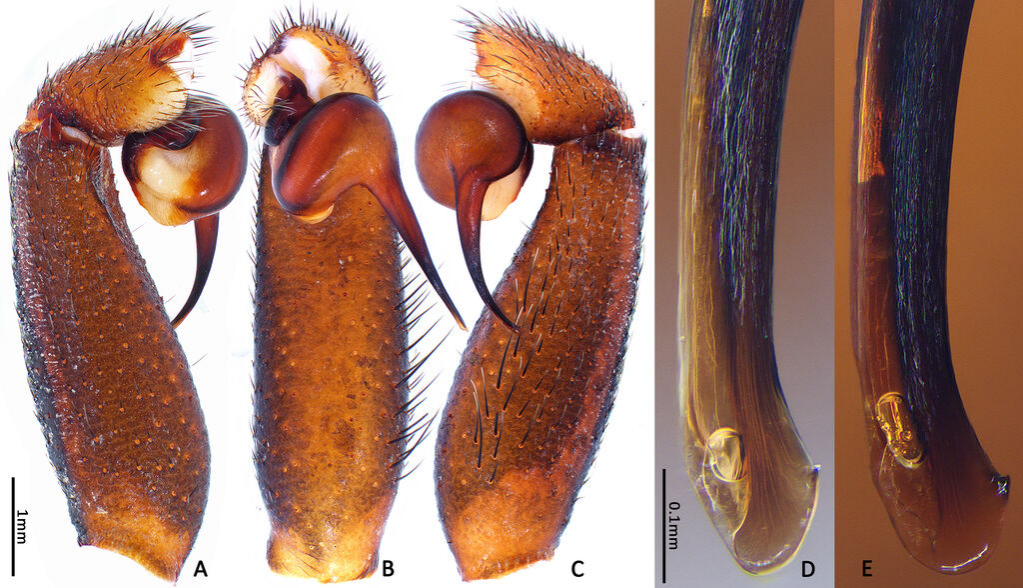
*Cyclocosmiaruyi* Yu & Zhang **sp. n.**, palp of paratype males. **A–C, E** MHBU-ARA-00023658; **D** MHBU-ARA-00023660; **D–E** Details of embolic tip in two individuals.

Embolus slender, tapering gradually into a fine tip that widens at its tip, to form a hook-like structure (Fig. 6D).

**Juveniles. First instar spiderling (Fig. 8D–F)**: body length 2.68 (not including chelicerae), carapace 0.96 long, 0.87 wide, abdomen oval, without opisthosomal disc, but three upper pairs of impressions (rows of impression I–III) and two lower unpaired transverse impressions (rows of impression IV–V) posteriorly (Fig. 8E–F); rastellum absent, front surface of chelicerae carrying one row of small spines. **Second instar spiderling (Fig. 8G–J)**: body length 3.17 (not including chelicerae), carapace 1.01 long, 0.96 wide, opisthosomal disc present, with ca. 40–45 radial ribs, carrying one bristle on each rib angle, upper muscle impressions kidney shaped (with elevated subcentral zone) (Fig. 8J); rastellum present, carrying one retrolateral-proximal spine and one distal spine (Fig. 8I). **Subadult male (penultimate)** like female in overall appearance and disc pattern, but palpal tarsi swollen proximally.

**Variation between adults (both sexes).** Count of radial ribs on female disc: 42–46 (n = 4); count of radial ribs on male disc: 44–48 (n = 2). Outlines of tip edges of embolus are slightly different in two males: relatively protracted in one, whereas relatively blunt in another one; apophysis of embolic tip show different sizes amongst two individuals (Fig. 6D–E). No obvious variation of embolic details observed between two palps in same individual.

#### Diagnosis

**Figure 7.**
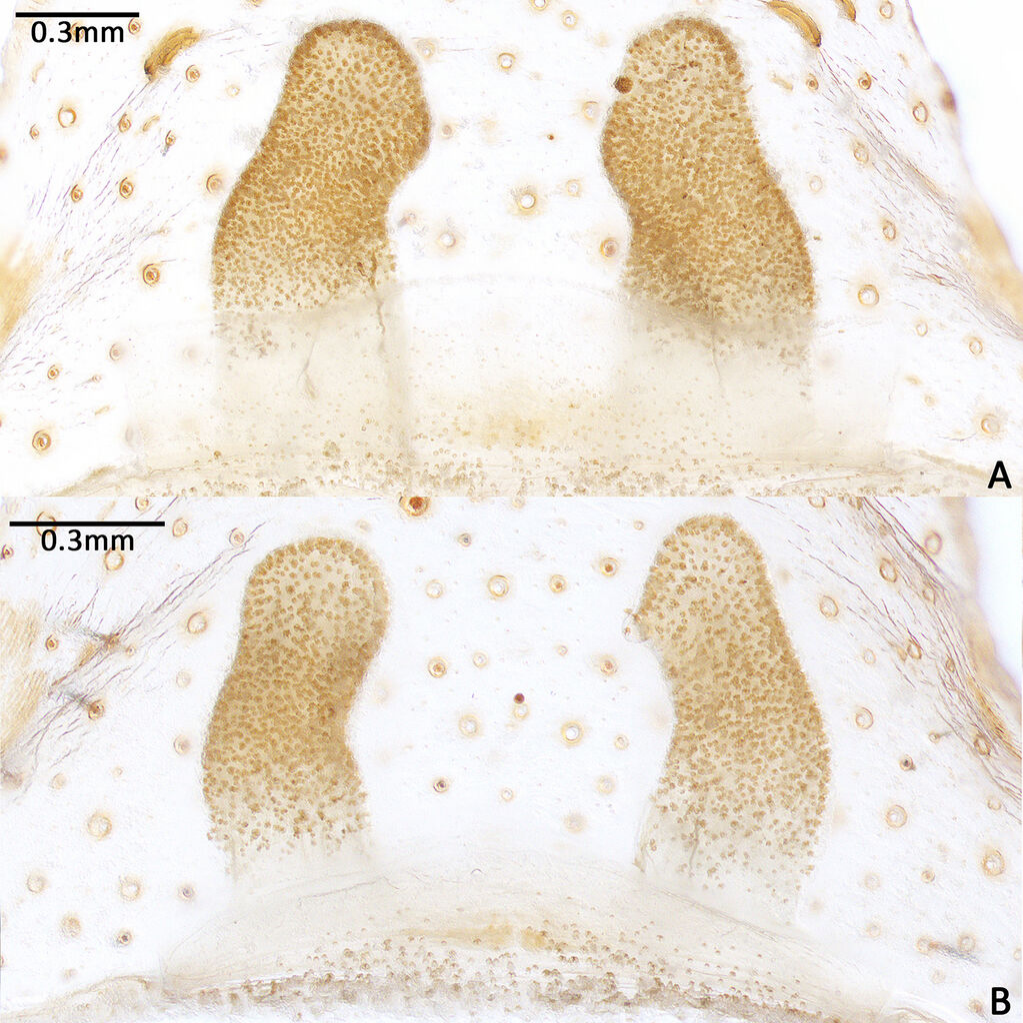
*Cyclocosmiaruyi* Yu & Zhang **sp. n.**, vulvae of females. **A** Holotype; **B** Paratype (MHBU-ARA-00023657).

The new species can be distinguished from the American congeners, *Cyclocosmialoricata* (C. L. Koch, 1842), *C.torreya* Gertsch & Platnick, 1975 and *C.truncata* (Hentz, 1841), by the more dense pores on spermathecae and the tips of spermathecae not processing lateral lobes (Fig. 7A–B, Fig. 9C–F), whereas the pores are relatively sparse and the lobes are well developed in the latter three species (see Gertsch and Platnick (1975): figs. 25–27).

In females, the new species can be distinguished from all Asian congeners (except for *C.lannaensis* Schwendinger, 2005) by the presence of tubercles on radial ribs (Fig. 5C–D) and spermathecae distally curved inwards (Fig. 7C–F, Fig. 9C–F). However, females can be distinguished from *C.lannaensis* by the following: (1) surface of radial ribs rather rough, with large number of small granulations (Fig. 12A); and (2) the lower transversal rib between upper and median muscle impressions is separated from radial ribs (Fig. 5C–D, 12A); whereas in *C.lannaensis*, surface of radial ribs is relatively smooth, without small granulations (Fig. 12B; Schwendinger (2005): fig. 51), the lower transversal rib between upper and median muscle impressions is connected to radial ribs (Fig. 12B; Schwendinger (2005): fig. 49). Further, females of the new species can also be distinguished from *C.siamensis* Schwendinger, 2005 by the absence of setae on the radial ribs (Fig. 5C–D), whereas setae are well developed on the radial ribs of disc in *C.siamensis* (see Schwendinger (2005): figs. 23–24). Females can be further distinguished from *C.ricketti* (Pocock, 1901) and *C.subricketti* Yu & Zhang, 2018 by: (1) the reduced number of radial ribs (count = 42–46); (2) absence of many small bristles on the clypeus (Fig. 9A), whereas in the latter two species, more than 60 radial ribs are present on the opisthosomal disc (Fig. 12C; Yu and Zhang (2018): fig. 5G) and many small bristles are present on clypeus (Fig. 9B; Schwendinger (2005): fig. 2). Females can be further distinguished from both *C.latusicosta* and *C.liui* Xu, Xu & Li, 2017 by the absence of an elevated central zone in either the upper or median pair of muscle impressions (Fig. 5C–D), whereas these are present in the upper muscle impressions of *C.latusicosta* forming endocentric concavities (Fig. 12D; Zhu et al. (2006): fig. 6B); or present in the median muscle impressions of *C.liui* (Xu et al. (2017): 83, fig. 3C).

In males, the new species can be distinguished from *C.latusicosta*, *C.ricketti*, *C.sublatusicosta* and *C.subricketti* by the following: (1) the presence of scopula on ventral tarsi IV (Fig. 10A); and (2) presence of more setae irregularly arranged on the dorsal abdominal ribs (Fig. 11A), whereas in *C.latusicosta*, *C.ricketti*, *C.sublatusicosta* and *C.subricketti*, the scopula is absent on ventral tarsi IV (Fig. 10B), the dorsal abdominal ribs have only a row of sparse setae only similarly dense distally (Fig. 11B). Males of the new species can be further distinguished from *C.ricketti*, *C.sublatusicosta* and *C.subricketti* by: (1) the reduced number of radial ribs (count = 44–48) (Fig. 5A–B); (2) in prolateral view, the apophysis of the embolic tip points laterally (Fig. 6D–E), whereas in *C.ricketti*, *C.sublatusicosta* and *C.subricketti*, more than 60 radial ribs are present on the opisthosomal disc (Lin et al. (2022): fig. 1D; Yu and Zhang (2018): figs 8B–C); while in prolateral view, the apophysis of the embolic tip points dorsally (Lin et al. (2022): fig. 5D). Males can be further distinguished from *C.latusicosta* by the absence of the elevated central zone in the upper muscle impression (Fig. 5A–B), whereas these are present in *C.latusicosta* (Fig. 12D; Yu and Zhang (2018): fig. 8A). Additionally, males can be distinguished from *C.lannaensis* and *C.siamensis* by the lower transversal rib between upper and median muscle impressions being separated from radial ribs (Fig. 5A), whereas in *C.lannaensis* and *C.siamensis*, the lower transversal rib between upper and median muscle impressions is connected to radial ribs (Schwendinger (2005): figs. 28, 53).

##### Remark

Interspecific genetic distances (*p*-distance) on COI sequences between *Cyclocosmiaruyi* sp. nov. and five other Asian congeners (*C.lannaensis*, *C.latusicosta*, *C.ricketti*, *C.sublatusicosta* and *C.subricketti*) form the range from 12.61% to 15.04%, which is considered comparable to interspecific genetic distances between *C.lannaensis*, *C.latusicosta* and *C.ricketti* (13.19% between *C.latusicosta* and *C.ricketti*; 14.84% between *C.lannaensis* and *C.latusicosta*; 17.09% between *C.lannaensis* and *C.ricketti*) and obvious higher than interspecific genetic distances between *C.ricketti*, *C.sublatusicosta* and *C.subricketti* (3.45 ~ 3.60% between *C.sublatusicosta* and *C.ricketti*; 6.30 ~ 6.45% between *C.ricketti* and *C.subricketti*; 6.60 ~ 6.90% between *C.subricketti* and *C.sublatusicosta*). Interspecific genetic distances between *C.ruyi* sp. nov. and two American congeners (*C.loricata* and *C.truncata*) form the range from 15.17% to 22.86%, considered comparable to interspecific genetic distances between other Asian *Cyclocosmia* species and American *Cyclocosmia* species (15.59 ~ 23.69%; Table 2).

#### Etymology

The specific epithet is from the Chinese "如意” (**rú yì**), it is an auspicious blessing, meaning “everything goes well”; noun in apposition.

#### Biology

All specimens were collected from moist slopes beside path cuts or hillside with abundant leaf litter (Fig. 1B). Burrows are usually with moss nearby. The entrance of burrow is closed by a thin trapdoor comprised of dead leaves, twigs and moss, sometimes with an extended rim (Fig. 1C–D). The burrow internally presents a wide upper and median portion (vestibule) and a constricted basal tube (Fig. 1E). Burrows of adult females are ca. 7–11 cm deep, shallower in juveniles.

Two mature males were obtained in early October, one matured in captivity from a subadult collected earlier (MHBU-ARA-00023660); another one was collected from the wild (MHBU-ARA-00023658). The collection of dust and soil on the body, shrunken abdomen and abrasion of some claws, indicate the latter one had perhaps been mature for some time. Two females were observed each carrying one egg sac on 3–4 August 2022. Daytime temperatures on those days ranged from a maximum of 30°C to a minimum of 20°C at night (perhaps relatively stable inside the burrow). When burrows were excavated, those females first tried to threaten by biting, before retreating to the constricted basal tube and blocking it with their abdominal disc, but leaving their egg sac behind themselves (Fig. 1F–G).

Two egg sacs were soon opened by the first author in the laboratory (10 August 2022). One of them had 147 live first instar spiderlings and same number of eggshells (Fig. 3A–B), the other had 158 fresh dead first instars, some eggshells and four eggs ready to hatch (this latter sac may have been ruined by accidental extrusion during carriage); no unfertilised eggs found. Living spiderlings were placed in a sunless incubator with 27°C and 95% humidity to nurture. After ca. 3–4 weeks (1–6 September 2022), they started to moult into second instars (Fig. 3C–E). The practical period of first instar stage is unknown, because the hatching time is uncertain, but presumably not too much longer.

**Figure 8.**
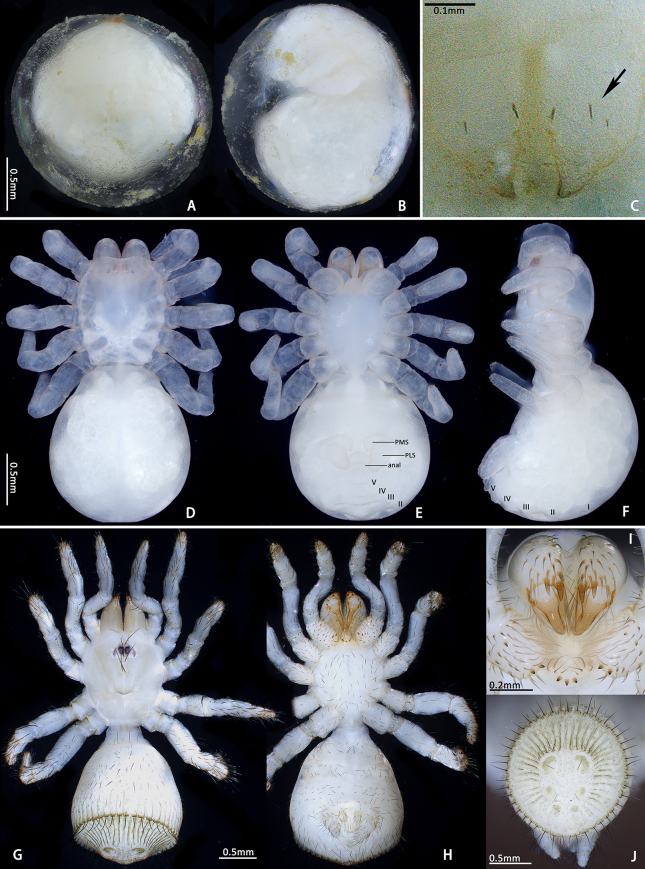
*Cyclocosmiaruyi* Yu & Zhang **sp. n.**, egg and early stages of spiderlings. **A–C** Pre-hatching egg; **D–F** First instar spiderling; **G–J** Second instar spiderling; **C** Chelicerae of embryo, with arrow indicating row of spines; **I** Chelicerae; **J** Opisthosomal disc. **A, C, I** Front; **B, F** Lateral; **D, G** Dorsal; **E, H** Ventral; **J** Back.

First instar spiderlings present a few of small spines on the front surface of the chelicerae, which do not seem homologous to those of the rastellum because of their deviation in position, arrangement and shape. These spines were developed before the hatching (Fig. 8C) and partly missing in some emerged first instars, which indicates they may be potentially used to puncture the eggshell. The opisthosomal disc was shaped up after moulting to second instar, three pairs of muscle impression were positionally corresponding to rows of impression II–IV in first instar; rows of impression I and V of first instar disappeared after moulting into second instar. These spiderlings seem very sensitive to light (even in early stage of first instar when their eyes were not well developed), they were very agitated under artificial lighting, but soon regained their sedentary composure after lighting was removed.

#### Distribution

**Figure 13.**
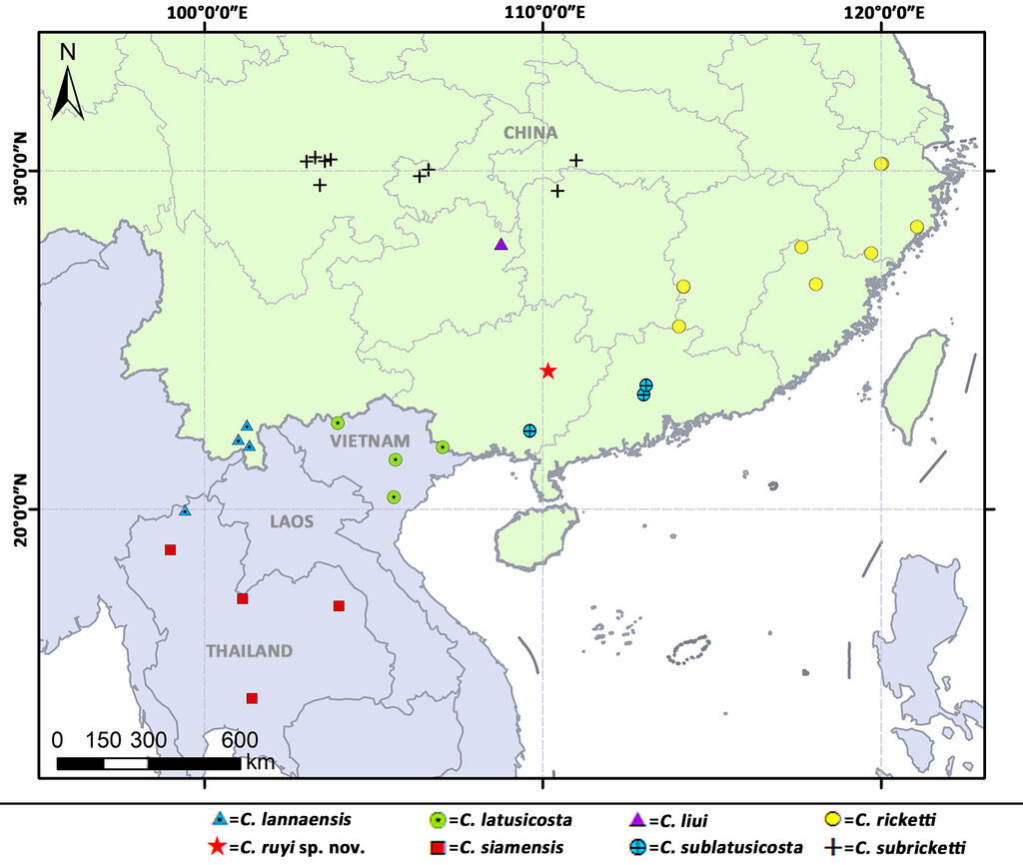
Distribution records of *Cyclocosmia* species in Asia.

Known only from the type locality of Guangxi, China (Fig. 13).

#### DNA barcode

AGATATTGGAACTCTTTATTTAATGTTTGGGGTTTGAGCTTCTATAATGGGTTCAGGTATAAGATTAATTATTCGAACTGAGTTAGGCCAATTAGGGAGATTTTTAGGTGATGATCATTTATATAATGTTATTGTGACAGCACATGCTTTAGTAATGATTTTTTTTATAGTGATGCCTATTATGATTGGGGGATTTGGAAATTGGTTGGTTCCTTTAATGATAGGGGCTCCAGATATAGCTTTTCCTCGGATGAATAATTTAAGATTTTGGTTATTGCCTCCTTCTTTGTTTATGTTGTTGCTTTCTTCTTTGACTGATTTAGGGGTAGGAGCTGGATGGACTATTTATCCTCCATTGTCTTCTTCTTTGGGGCATATAGGGGGGGGGATAGATTTTGTTATTTTTTCTTTGCATTTGGCAGGGGCTTCTTCAATTATAGGGGCTATTAATTTTATTTCAACTATTGTGAATATACGATCTTCTGGAATGAGTTTGGAACGAGTTCCTTTGTTTGTGTGATCTGTGATGATCACAGCTATTTTATTGTTATTGTCGTTACCAGTTTTAGCTGGAGCGATTACTATATTGTTGACTGACCGGAATTTTAATACTTCTTTTTTTGATCCTGCTGGAGGAGGAGATCCTATTTTATTTCASCATTTATTTTGATTTTTTGGTC **(GenBank accession number: OQ561524)**

## Supplementary Material

XML Treatment for Cyclocosmia ruyi

## Figures and Tables

**Figure 9a.**
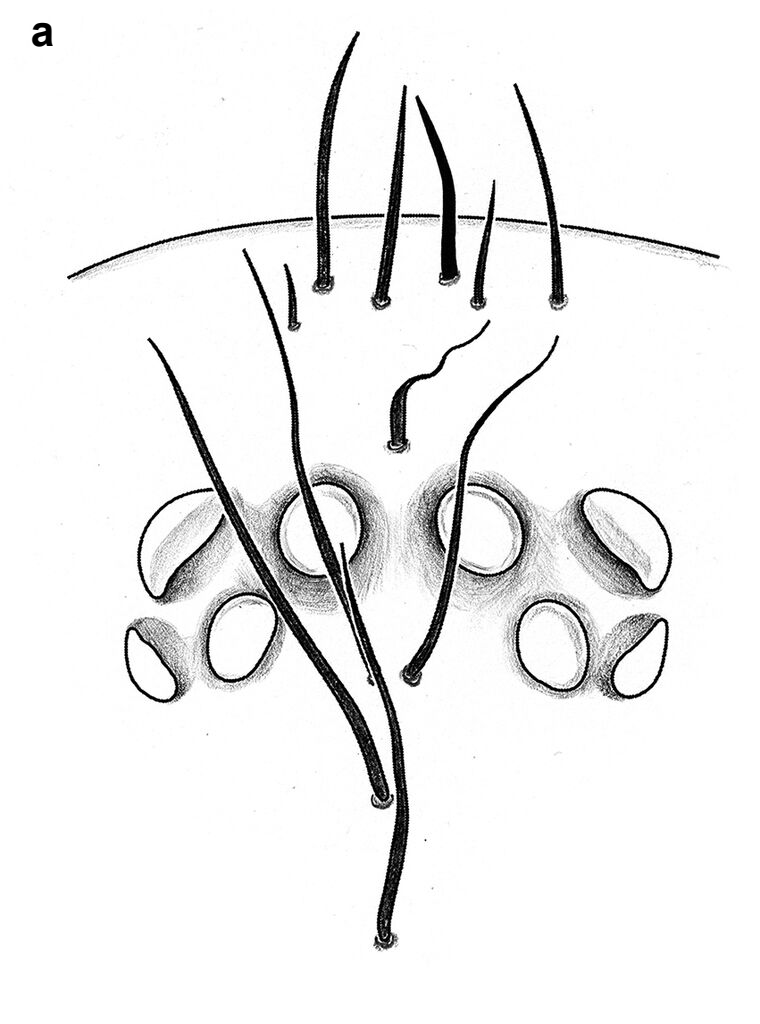
*C.ruyi* Yu & Zhang, **sp. n.**, paratype female (MHBU-ARA-00023659);

**Figure 9b.**
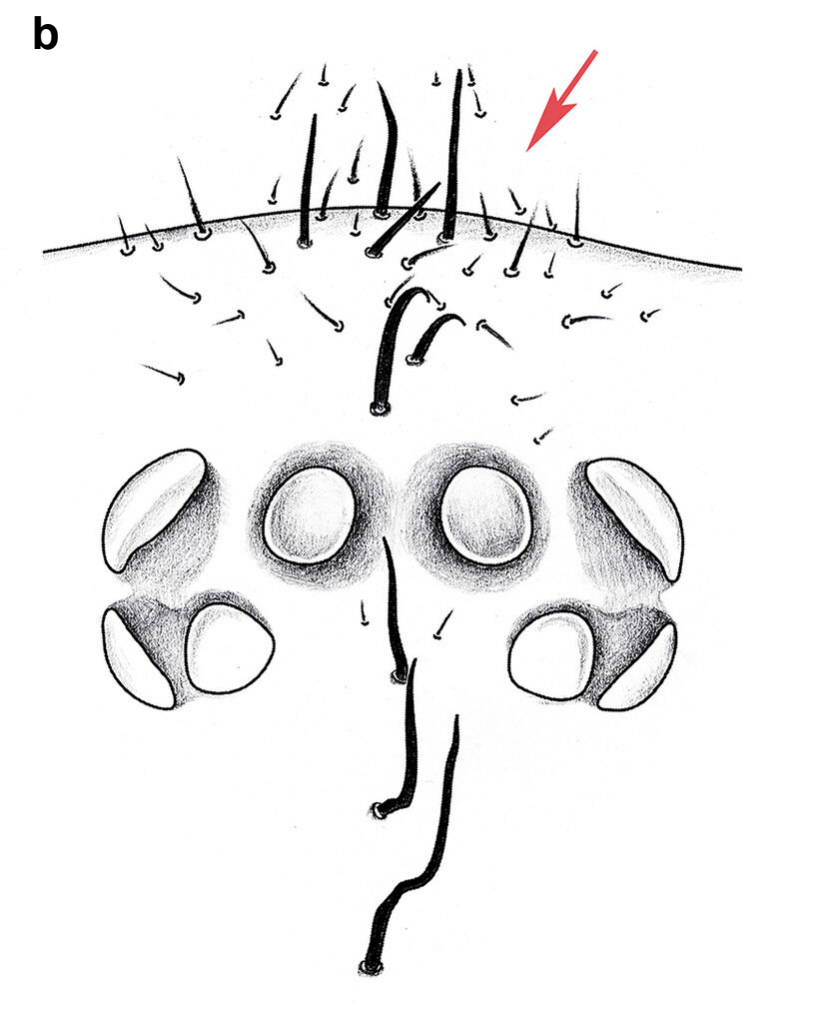
*C.subricketti* from Pujiang County, Sichuan Province (CKYH), with arrow indicating dense small bristles;

**Figure 9c.**
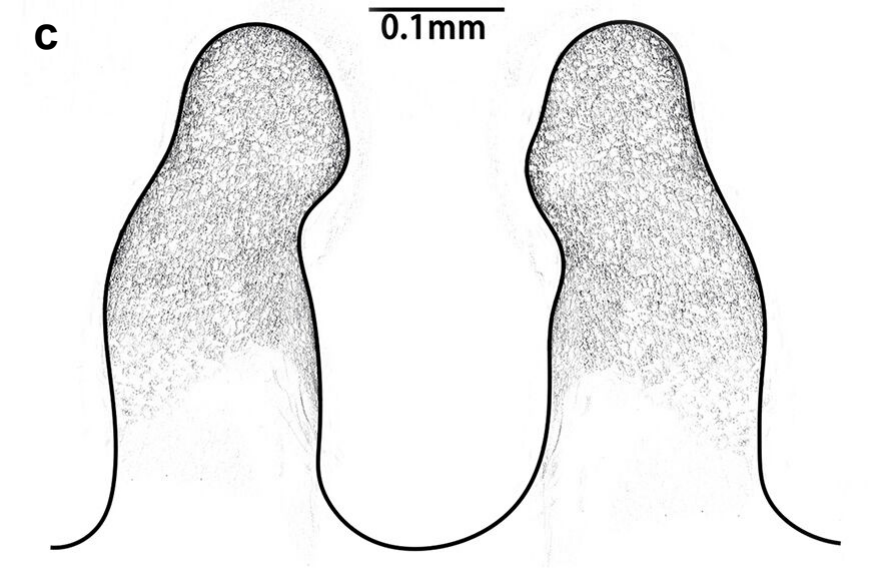
Holotype;

**Figure 9d.**
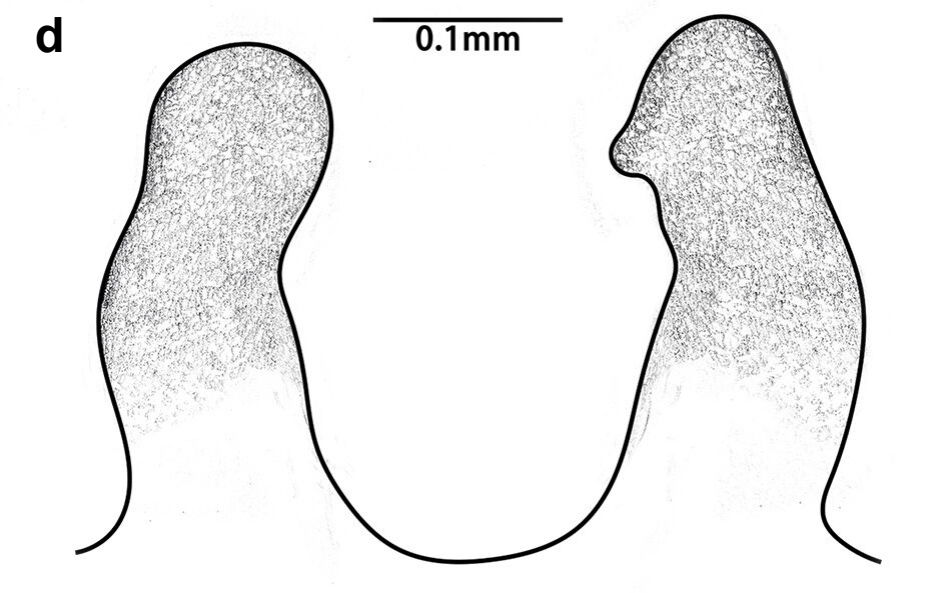
Paratype (MHBU-ARA-00023657);

**Figure 9e.**
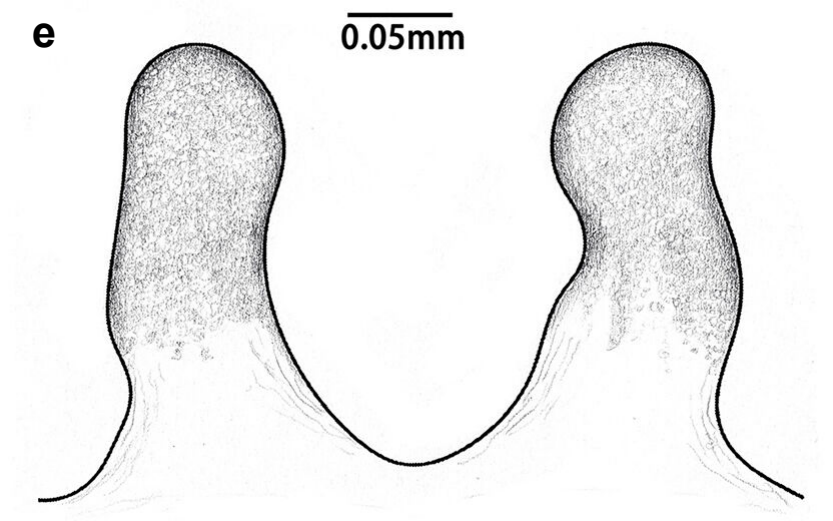
Paratype (MHBU-ARA-00023659);

**Figure 9f.**
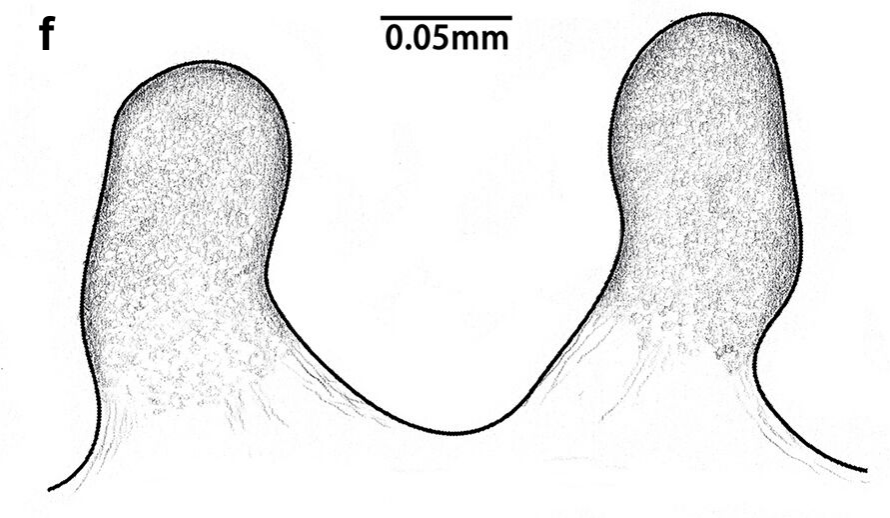
Non-type specimen, subadult female (CKYH).

**Figure 10a.**
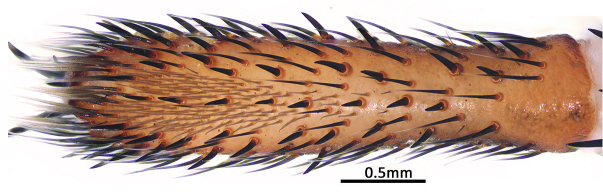
*C.ruyi* sp. nov., paratype (MHBU-ARA-00023660);

**Figure 10b.**
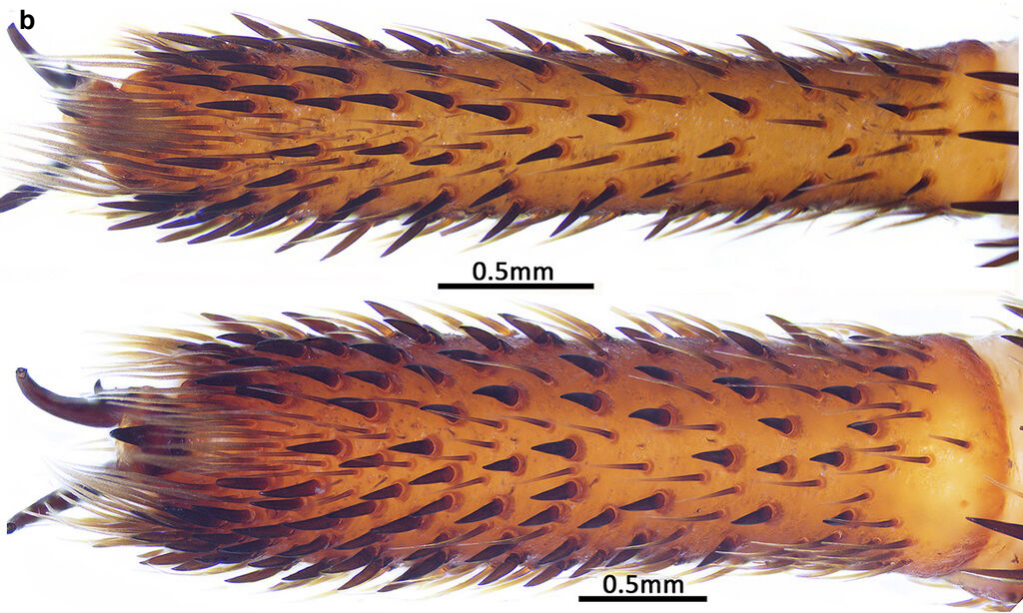
*C.ricketti* from Hangzhou, Mt. Xiaohe (upper; CKYH) and *C.latusicosta* from northern Vietnam (lower; CKYH).

**Figure 11a.**
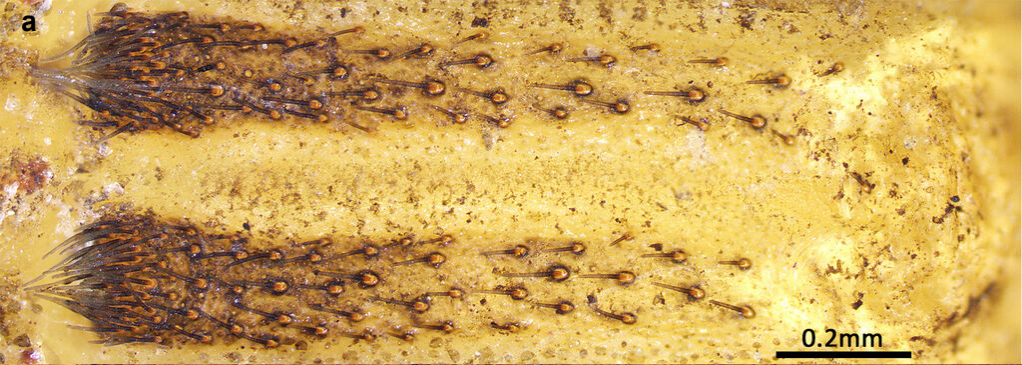
*C.ruyi* sp. nov., paratype (MHBU-ARA-00023660);

**Figure 11b.**
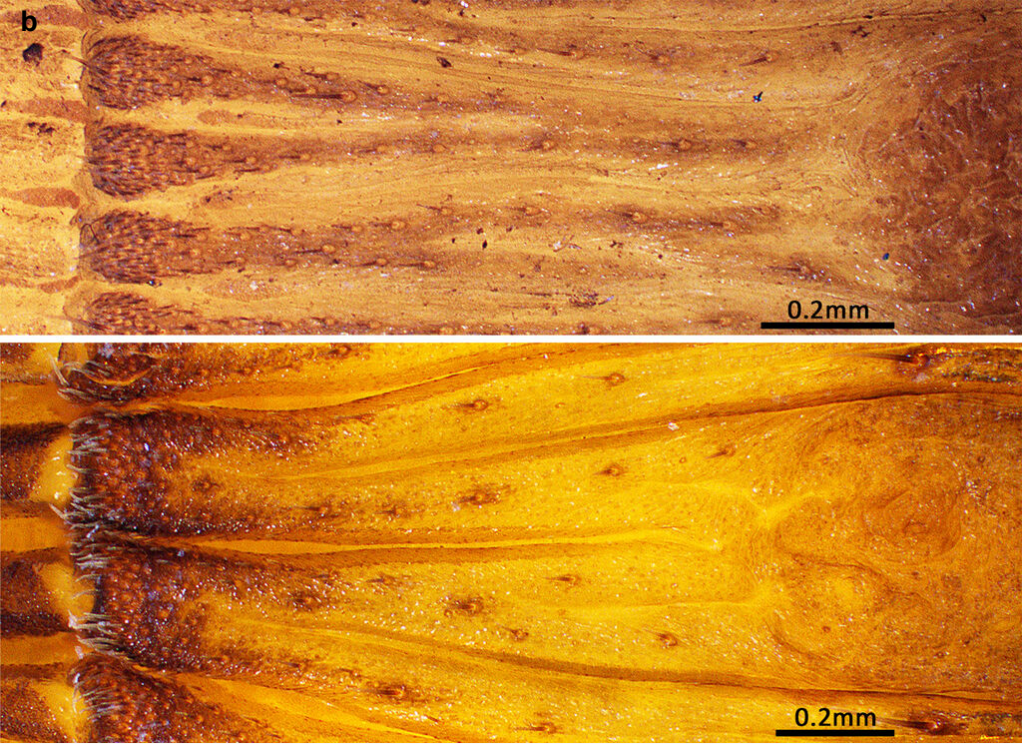
*C.ricketti* from Hangzhou, Mt. Xiaohe (upper; CKYH) and *C.latusicosta* from northern Vietnam (lower; CKYH).

**Figure 12a.**
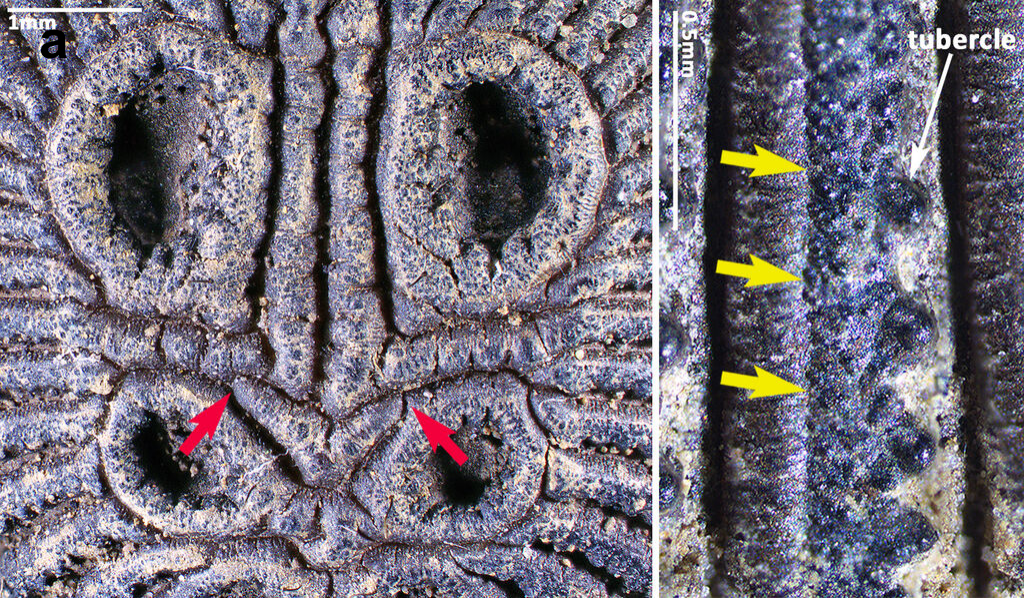
*C.ruyi* sp. nov., Paratype females (left: MHBU-ARA-00023657, with red arrows indicating the ends of the lower transversal rib between upper and median muscle impressions; right: MHBU-ARA-00023659, details of radial rib, with yellow arrows indicating the rough surface with large number of granulations);

**Figure 12b.**
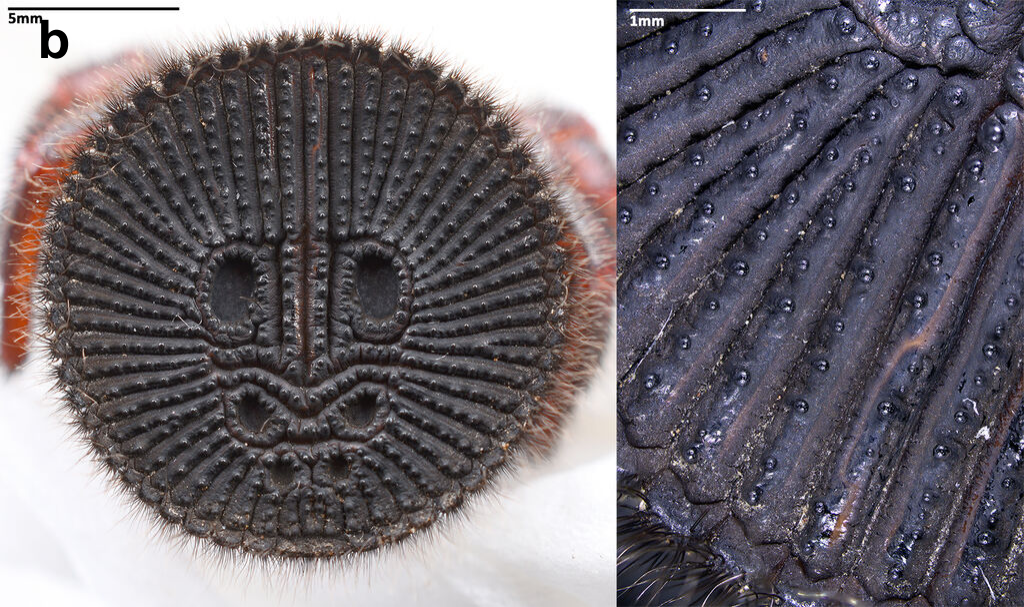
*C.lannaensis*, female from Xishuangbanna, China (CKYH); left: opisthosomal disc; right: details of radial ribs;

**Figure 12c.**
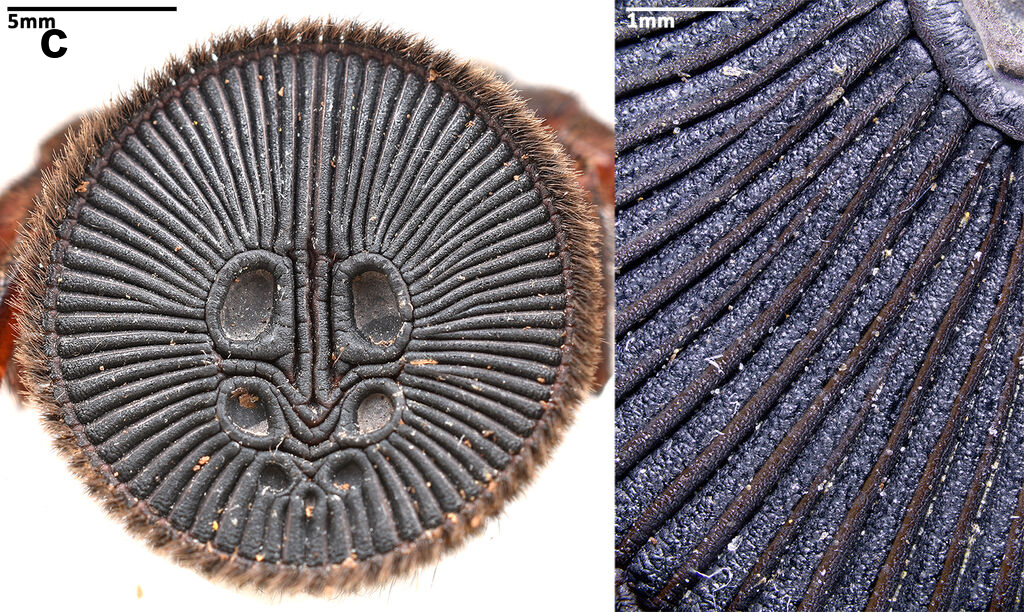
*C.ricketti*, female from Jiangxi Province, China (CKYH), left: opisthosomal disc; right: details of radial ribs;

**Figure 12d.**
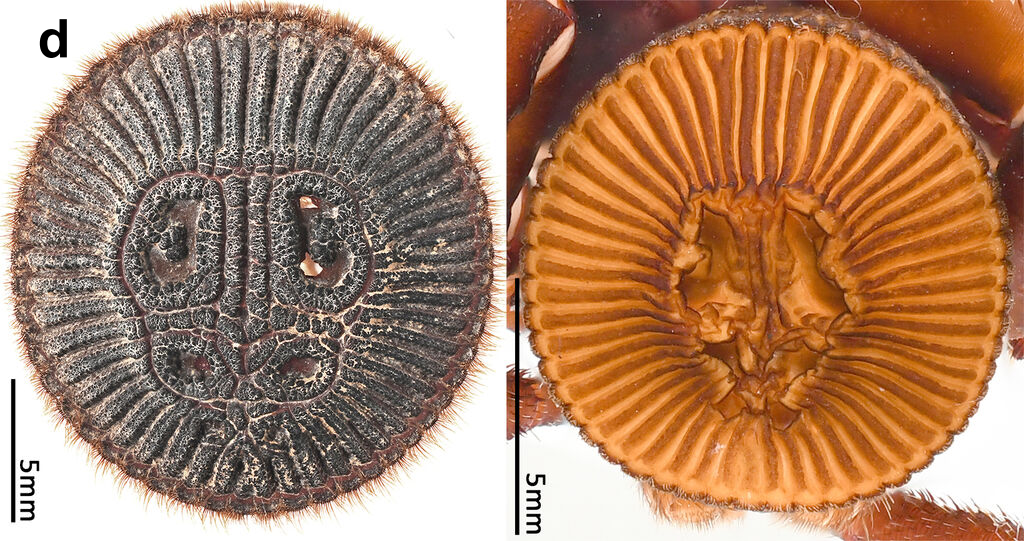
*C.latusicosta*, opisthosomal discs, left: paratype female from Guangxi, China (MHBU); right: male from northern Vietnam (CKYH).

**Table 1.** List of GenBank accession numbers, voucher numbers and locations of *Cyclocosmia* specimens used for molecular comparison in this study.

**GenBank Accession Number**	**Taxon**	**DNA Voucher number**	**Location**
OQ561523	*C.ruyi* sp. nov.	KYU081	CHINA: type locality in Guangxi Prov.
OQ561524	*C.ruyi* sp. nov.	KYU082	CHINA: type locality in Guangxi Prov.
OQ561525	*C.lannaensis*	KYU083	CHINA: Yunnan Prov., Xishuangbanna, Menglun Town
OQ561526	*C.latusicosta*	KYU084	VIETNAM: near Lào Cai
OQ561527	*C.ricketti*	KYU072	CHINA: Zhejiang Prov., Hangzhou, Lishanqiao Village
OQ572318	*C.ricketti*	KYU071	CHINA: Zhejiang Prov., Wenzhou, Mt. Yandang
OQ572316	*C.sublatusicosta*	KYU065	CHINA: Guangdong Prov., Foshan, Changqi Village
OQ572317	*C.sublatusicosta*	KYU073	CHINA: Guangxi Prov., Qinzhou, Pubei County
OQ572319	*C.subricketti*	KYU007	CHINA: Sichuan Prov., Pujiang County
OQ572320	*C.subricketti*	KYU079	CHINA: Chongqing, Jindao Gorge
OQ572322	*C.subricketti*	KYU070	CHINA: Hubei Prov., Yichang, Duzhenwan Town
OQ572321	*C.subricketti*	KYU067	CHINA: Sichuan Prov., Mt. Emei
KY017640.1	*C.truncata*	AUM MY2033	USA: Alabama, Lawrence Co., Borden Ck. Trail
KY017639.1	*C.loricata*	AUM MY3547	MEXICO: Nuevo Leon, El Potosi

**Table 2.** Interspecific genetic distance matrix (based on *p*-distance model) on COI sequences of eight species of *Cyclocosmia*.

	OQ561523	OQ561524	OQ561525	OQ561526	OQ561527	OQ572318	OQ572316	OQ572317	OQ572319	OQ572320	OQ572322	OQ572321	KY017640.1	KY017639.1
OQ561523														
OQ561524	0.0030													
OQ561525	0.1278	0.1261												
OQ561526	0.1368	0.1366	0.1484											
OQ561527	0.1504	0.1502	0.1709	0.1319										
OQ572318	0.1504	0.1502	0.1709	0.1319	0.0000									
OQ572316	0.1414	0.1411	0.1619	0.1244	0.0345	0.0345								
OQ572317	0.1383	0.1381	0.1589	0.1229	0.0360	0.0360	0.0045							
OQ572319	0.1383	0.1351	0.1619	0.1244	0.0630	0.0630	0.0660	0.0675						
OQ572320	0.1383	0.1351	0.1619	0.1244	0.0630	0.0630	0.0660	0.0675	0.0000					
OQ572322	0.1383	0.1351	0.1619	0.1229	0.0645	0.0645	0.0675	0.0690	0.0015	0.0015				
OQ572321	0.1383	0.1351	0.1619	0.1244	0.0630	0.0630	0.0660	0.0675	0.0000	0.0000	0.0015			
KY017640.1	0.1549	0.1517	0.1559	0.1664	0.1784	0.1784	0.1739	0.1724	0.1649	0.1649	0.1664	0.1649		
KY017639.1	0.2286	0.2252	0.2249	0.2369	0.2369	0.2369	0.2294	0.2279	0.2264	0.2264	0.2279	0.2264	0.2264	
